# Happy Old Age

**Published:** 1921-05-28

**Authors:** 


### Happy Old Age.

There is a limited number of medical and surgical optimists who have recently been exciting the public mind in all countries by suggesting experimental methods by which the old may grow young. By way of a corrective we should like to draw the attention of our readers to some prudent remarks made last week by Sir G. Sims Woodhead in which he showed how it is possible to grow old and to be happy in old age. Sir Sims Woodhead, who is Principal of the Medical Schools at Cambridge University, told the People's League of Health that if you take, as Sir George Humphry did, a whole series of old people you will find that they lived under very good natural conditions. Sixty or seventy per cent. of them were not over-active, but they took plenty of exercise. When they were young they were not overworked. Their mental development was carried on slowly; their physical development was also carried on comparatively slowly, but equally throughout. They did not over-eat or over-drink.

The first thing to notice about old people is how extraordinarily healthy they are. You will find some people at fifty have hard vessels, but the very old man seldom has a hard vessel. These people, in brief, have lived to be old because they are healthy. A few of them show signs of age, but these are the people in whom there is some disease, and it is not in old age that we get unhealthy tissues. In the healthy individual person the processes of death take place so equally that no single part of the body gives way before the other. That is the perfection of old age. The simplicity of its perfection is remarkable.

